# The Journey of Artificial Intelligence in Food Authentication: From Label Attribute to Fraud Detection

**DOI:** 10.3390/foods14101808

**Published:** 2025-05-19

**Authors:** Dana Alina Magdas, Ariana Raluca Hategan, Maria David, Camelia Berghian-Grosan

**Affiliations:** 1 National Institute for Research and Development of Isotopic and Molecular Technologies, 67-103 Donat Street, 400293 Cluj-Napoca, Romania; ariana.hategan@itim-cj.ro (A.R.H.); maria.david@itim-cj.ro (M.D.); camelia.grosan@itim-cj.ro (C.B.-G.); 2 Faculty of Physics, Babeș-Bolyai University, Kogălniceanu 1, 400084 Cluj-Napoca, Romania

**Keywords:** food authentication, processing strategies, artificial intelligence, food adulteration, image processing

## Abstract

Artificial intelligence (AI) tends to be extensively used to develop reliable, fast, and inexpensive tools for authenticity control. Initially applied for food differentiation as an alternative to statistical methods, AI tools opened a new dimension in adulteration identification based on images. This comprehensive review aims to emphasize the main pillars for applying AI for food authentication: (i) food classification; (ii) detection of subtle adulteration through extraneous ingredient addition/substitution; and (iii) fast recognition tools development based on image processing. As opposed to statistical methods, AI proves to be a valuable tool for quality and authenticity assessment, especially for input data represented by digital images. This review highlights the successful application of AI on data obtained through laborious, highly sensitive analytical methods up to very easy-to-record data by non-experimented personnel (i.e., image acquisition). The enhanced capability of AI can substitute the need for expensive and time-consuming analysis to generate the same conclusion.

## 1. Introduction

Because of the significant natural variability of food commodities and also the insertion on the market of products that have passed many times through a subtle and hard-to-detect adulteration process, the development of reliable strategies for counterfeit detection represents a challenge and involves a huge amount of data impossible to handle by human processing capabilities alone. For this reason, the involvement of supervised statistical tools and, more recently, artificial intelligence (AI) for the development of reliable instruments for the detection of sophisticated adulterations has become an important strategy. The increased demand for the application of the above-mentioned data processing strategies is in strong connection with the new tendency regarding the application of rapid and green analytical methods, as they are simultaneously encouraged by the development of portable market equipment [1]. Moreover, during recent years, the enhanced capabilities of AI to extract subtle and meaningful information from a huge amount of data and to generate well-grounded assessments has transcended limits with the development of reliable tools for food quality and authenticity verdicts based on digital images, hyperspectral data, thermographic images, etc. [2,3].

The initial use of AI for the development of authentication tools went hand in hand with advanced supervised statistical strategies, as they were used as an alternative to the latest or for validation purposes. This is because, in this early stage, AI was especially applied for the development of classification models for food differentiation with respect to several label attributes, like the geographical origin, botanical source, animal species, or production year. It was proven through several studies that the application of either advanced statistical methods or AI resulted in the achievement of comparable recognition models in terms of accuracy [4,5]. Afterwards, the potential of AI for food fraud detection— even for common frauds, like the addition of sugar in fruit juices [6], or for ones that are very subtle and difficult to detect, as is the case of partial substitution of a food variety with a cheap one, like undeclared oil or honey mixtures [7,8,9]—was recognized and transformed through effective tools. During the last few years, a step forward was made through employment of the enhanced capabilities of AI to interconnect the provided data and to construct recognition models based on images, with these provided data recorded through analytical procedures or derived from visual files [10,11].

The recognized applications of AI in the development of reliable tools for food authentication, given the provided advantages in terms of rapidity, in which a huge volume of data can be processed and interconnected to extract meaningful conclusions in the entire field of food production and control, have led to the investigation of this potential by distinct research groups. Many of these efforts were recently acknowledged in well-documented review papers [1,2,12,13] in which several aspects of the potential of using AI in the food industry were presented. Notably, the work of Medina et al. highlighted the efficiency of AI-based methods in corroboration with recently introduced rapid and non-invasive analytical methods (e.g., spectroscopic techniques) in comparison with state-of-the-art analytical approaches [1].

The published papers that present the limitations of the current chemometric approaches and highlight the great emerging potential of applying deep learning (DL) combined with spectroscopic techniques for quality evaluation in terms of variety identification, geographical origin detection, and adulteration recognition of food and agro-products were summarized by Zhang and co-workers [12]. In their view, the benefits of DL arise from the independence from human input and from the improved precision and large-scale applicability. Another research group discussed, based on the published data in the literature, the great potential of using DL as a data analysis tool in quality detection and recognition in the food domain, indicating that it outperforms conventional learning-based methods and has the ability to automatically generate features that are better than the handcrafted ones, but it has limitations, such as long training times due to the large size of the original data [3].

Meenu et al. assessed the recent advancements in using digital image processing (DIP) for predicting the quality of various food products as a need to increase commercial exploitation, which cannot be achieved through classic computer vision algorithms [2]. In their view, the development of mobile applications integrating DIP tools based on DL offers new opportunities in food control. The application of convolutional neural networks (CNN) as deep feature extractors for effectively and efficiently detecting and analyzing complex food matrices was presented and discussed in another review paper that pointed out the improvements that such deep learning methods can bring over conventional machine learning (ML) algorithms, presenting the benefits of applying them in future studies for food detection and analysis [13].

**Figure 1:**
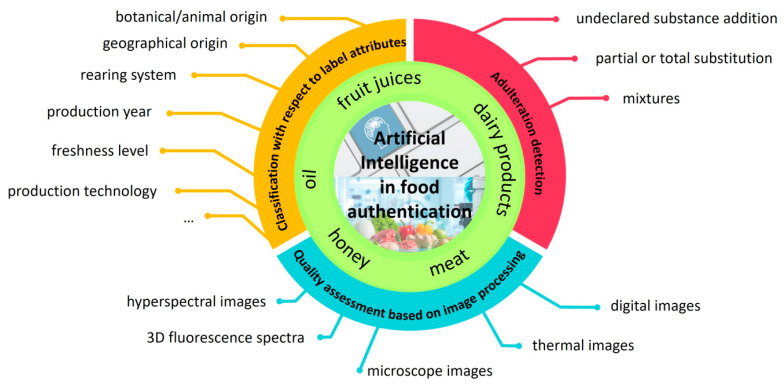
Preview of the main applications of AI in food fraud control.

Against the already published review papers, the aim of the present comprehensive review was to synthesize the reported results in the literature in which AI was applied to support the food chain, starting from the incipient purposes for which the applicability of AI was prospected until nowadays, when a new dimension in the food control field is opening based on the enhancement of data interconnections provided by AI. Thus, this review is structured to point out the main contributions of AI in the development of reliable tools for food control regarding (i) food differentiation with respect to different label characteristics; (ii) adulteration detection through total or partial substitution; and (iii) development of fast recognition tools for the food industry based on image processing (Figure 1).

For this purpose, we conducted a comprehensive literature search utilizing the Web of Science and Google Scholar databases. The primary aim was to provide an overview of the application of AI to solve several problems that occur along the food chain, like the false declaration of label attributes (e.g., geographical, botanical or animal origin, production year, production and condition technologies, and freshness level) or adulterations with extraneous substances or cheaper varieties, and also to compare the efficiency of AI with that provided by classical chemometric approaches in terms of similarities, advantages, and limitations when applicable.

Based on these premises, an in-depth literature search was undertaken using various keyword combinations. More specifically, on the Web of Science platform, our initial query was defined as T = (food AND (artificial intelligence OR ai OR machine learning OR ml OR deep learning OR dl OR statistical OR chemometrics) AND (classification OR authenticity OR adulteration)), where T refers to the title, author keywords, or abstract tags.

Because of the available literature distribution concerning the application of AI in food control and also based on the assumed main directions we followed, five food matrices are discussed in this work. These are honey, oil, fruit juices, dairy products, and meat. It was not surprising that all matrices that benefited from the highest interest from researcher groups belong to the ten food commodities most susceptible to fraud, according to a report published by the European Parliament [14].

After the selection of the matrices of interest, the search domain was restricted based on additional keywords, such as the food commodities’ names (e.g., honey) or analytical technique names.

## 2. A General Overview of AI in Food Authenticity Assessment

With a widely recognized applicability in numerous fields owing to its capacity in improving automation, productivity, and connectivity and with its important role within Industry 4.0, AI has also become, in recent years, an increasingly utilized technology in food authenticity assessment [15,16]. Its ability to process large volumes of complex, multivariate data and to extract meaningful markers and patterns has made it particularly suitable for numerous applications, such as origin verification or adulteration detection for various food matrices, facilitating efficient, rapid, non-destructive, and on-site investigations for solving fraud problems.

At a theoretical level, AI refers to a domain within the general field of computer science that comprises computational systems designed to mimic human cognitive functions such as learning, acting logically, reasoning, or problem solving [17]. Within the scope of food authenticity assessment, AI is most often implemented through ML algorithms, which refer to the development of system models that learn, from labeled or unlabeled training datasets, to perform specific tasks through a process that implies the improvement of their performance by experience [18]. In supervised learning, the model is trained using labeled input data that enables generalization, which subsequently allows the prediction of unknown input instances [18]. Algorithms such as artificial neural networks (ANN), support vector machines (SVM), random forests (RF), or classification and regression trees (CART) have demonstrated high applicability in classifying food products according to distinct label attributes or in detecting and quantifying adulteration. Unsupervised learning, by contrast, implies the development of descriptive models that identify specific patterns in completely unlabeled training data [19]. Such algorithms (e.g., clustering methods, PCA, or self-organizing maps—SOMs) are often employed to detect groupings of samples without prior knowledge of class memberships or to identify outliers.

DL, a branch of ML, has emerged along with the increasing availability of computational resources, which has facilitated a transition from traditional learning methods based on linear and kernel techniques to more complex neural structures, often referred to as deep neural networks. These networks can automatically extract relevant features from experimental data and effectively model nonlinear relationships, leading to superior performance in diverse prediction tasks. As a result, DL was found to be particularly suitable for several applications in the field of food authentication that involve complex data such as images or spectroscopic fingerprints [20,21].

The reliability of AI-based models developed for specific applications in the field of food analysis is dependent on a rigorous evaluation of their performance. In the context of supervised learning, this procedure usually begins with the split of the dataset into training and testing sets: the training instances are used for constructing the model, while the test samples allow an unbiased assessment of the performance on unseen data. In the case when there is a limited number of investigated samples and when the allocation of a subset of samples solely for testing purposes affects the training or testing data representativeness, cross-validation is commonly employed as an evaluation and model optimization strategy. This involves partitioning the dataset into multiple subsets and iteratively testing each one of them through the development of multiple prediction models [22].

To assess the effectiveness of the models constructed, several metrics can be computed. For binary classification tasks, performance measures are usually represented by accuracy (i.e., the proportion of correctly predicted samples); precision (i.e., the proportion of true-positive samples among the positive predicted samples); sensitivity, frequently also referred to as recall (i.e., the ratio of true positives to all positive samples); specificity (i.e., the ratio of true negatives to all negative samples); F1-score (i.e., the harmonic mean of precision and recall); and the area under the receiver operating characteristic curve (AUC), with each offering insights into different aspects of performance, especially in the case of class imbalance [23]. In the case of regression problems, the most commonly used metrics comprise the mean absolute error (MAE) or the mean squared error (MSE) [23].

## 3. AI as an Effective Tool for Food Classification

The employment of advanced statistical methods in the development of reliable tools for food differentiation represented a step forward in the food industry, opening new possibilities to apply spectroscopic techniques in the construction of reliable differentiation models [24]. This is because of the very subtle differences that occur from one sample to another when rapid spectroscopic techniques are used, such as UV-Vis, vibrational, NMR, etc. Due to these small differences, a characteristic pattern for the samples belonging to the same group cannot be observed or identified by the human eye, but it can be easily pointed out using statistical methods. Therefore, due to the involvement of statistical tools, it became possible to successfully apply, for food authentication purposes, analytical techniques that are fast, cost-effective, and require a simple or no preparation step. In this way, the application of green analytical techniques in this field took a step forward. Statistical methods have been extensively employed in the literature for the development of food classification models, obtaining high accuracies and being useful in authenticating food matrices, as shown in Table 1. Even if the statistical methods opened the door for the so-called “foodomics”, in parallel, in the last 20 years, with noticeable increases in the last few years, more and more studies involving the application of AI for classification purposes have been reported (Table 1) [12,25]. The analyzed food matrices in the frame of the present study, namely honey, oil, fruit juices, dairy products, and meat, are very complex. Therefore, the detection of subtle compositional differences is difficult to observe, and statistical tools or AI models can be employed to process large datasets to distinguish and classify authentic samples.

**Figure 2:**
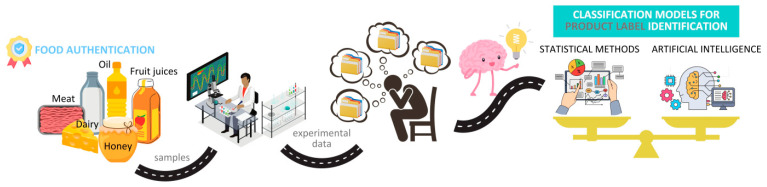
Comparable performances between the use of advanced statistical methods and AI for food recognition models development.

As stated by many authors [4,5], the classification model accuracies were not significantly improved through the substitution of statistical methods with AI but rather by a validation of the reliability of the proposed approaches through the achievement of comparable results when the two previously mentioned data strategies were used (Figure 2). Moreover, as each food matrix has its own particularities and has been explored in the literature through several points of view, separate attention is given in the present paper to each discussed food commodity in order to better understand the role and which strategy is the most appropriate for use.

### 3.1. Honey

Honey is a very complex matrix, containing over 200 compounds, whose concentrations can slightly vary and have been proven to have natural variability based on the botanical and geographical source. The authentication issue is very important, as monofloral honeys have an increased market value. Because of the matrix complexity, traditional chemical profiling may not reveal subtle compositional differences. Therefore, statistical tools and AI models, particularly those using spectroscopic or chromatographic data, can learn complex nonlinear patterns in large datasets to distinguish authentic samples. Numerous classification tools, relying on the information achieved through various analytical techniques, have been reported in the literature for identifying the origin of honey. According to a recent review performed by Tsagkaris et al. [39], among these studies, the majority aimed at the development of classification models for botanical and geographical origin assessment, while the most widely applied analytical techniques corresponded to chromatography, physiochemical analysis, spectroscopic techniques, isotopic and elemental determinations, and sensory analysis. As analytical techniques such as chromatographic analysis do not require the application of advanced data processing methods, studies implying the use of isotope and elemental determinations or distinct spectroscopy techniques in corroboration with statistical and, more importantly for the aim of our review, AI are further discussed.

Isotope ratios have become a widely recognized method for assessing the honey’s geographical and botanical provenance [40,41]. Apart from the isotope fingerprint, the multi-elemental content of honey has been reported in several research studies [42,43] as valuable information for characterizing the varietal and geographical source of honey. The majority of the studies that have relied on stable isotope ratios or multi-element determinations illustrated the use of statistical techniques like discriminant analysis [44], soft independent modeling of class analogy (SIMCA) [41], multivariate analysis of variance [45], or partial least squares discriminant analysis (PLS-DA) [46] for constructing honey prediction models. However, there have also been studies that proposed the application of AI for this task. In this context, one of the earliest of such studies is the work of Batista et al., in which the application of SVM, random forest (RF), and multilayer perceptron was investigated for recognizing honey harvested in São Paulo [47].

The successful application of ANN for geographically differentiating honey based on the isotope and elemental profile was also investigated in the study of Hategan et al. [48], which aimed to classify honey with respect to the country of provenance. Chen et al. also tested the potential of ANN for classifying honey belonging to four varieties, namely acacia, linden, colza, and vitex, based on 10 elemental concentrations and highlighted the efficiency of the ANN model as compared to a statistical treatment performed through PLS-DA [46]. The potential given by the association of the elemental composition and ML for classifying honey with respect to the botanical origin was investigated by Karabagias et al. [45], who reported a 78.9% accuracy score for this task. A recent research study published by Liu et al. indicated the effectiveness of applying RF for honey botanical discrimination based on the association of the isotopic fingerprint and element composition [26]. In this regard, a much higher accuracy of 96% in predicting honey samples of six botanical varieties was achieved by the RF model during the testing phase. The reported results also highlighted that higher classification performances are obtained by applying RF in comparison to SVM or statistical methods like linear discriminant analysis (LDA).

Currently, attempts are being made to replace analytical methods that require a long preparation time, are expensive, and therefore involve considerable financial and human effort with faster and more cost-effective alternatives [29]. In this regard, there is a desire to employ analytical techniques that are based on various types of spectroscopy, like IR [27,49,50], Raman [29,51], fluorescence [52,53], and NMR [54,55], allowing the classification of samples with a precision similar to or even higher than that obtained by traditional analytical techniques. As data processing strategies, statistical methods seem to be a more frequent choice compared to AI-based approaches [56], which also benefited from increased attention when these analytical techniques were employed [28,29,49,52,57,58]. While some studies have highlighted the efficiency of AI-based algorithms such as SVM over PLS-DA [28] for the botanical origin assessment and over LDA for honey classification according to the geographical origin [57], a general conclusion cannot be stated, as there are studies proving the contrary. For example, in the work of Magdas et al. [29], an indirect comparison between the ability of statistical (i.e., SIMCA) and ML techniques (i.e., bagged trees ensemble, SVM, etc.) in discriminating honey samples with respect to the geographical and botanical origin based on Raman spectroscopy was conducted, highlighting that SIMCA modeling slightly outperformed the ability of learning-based strategies for this specific task.

Based on the previously described literature overview concerning honey origin authentication, an overall conclusion regarding the efficiency of AI-based approaches over the performance of statistical methods could not be reached.

### 3.2. Oils

Edible oils are frequently subjected to authentication studies, as it is critical to verify the origin of an oil in order to justify its selling price, verify its beneficial properties, and identify the markers specific to each oil type to discourage fraudulent practices. For the edible oil market, a significant issue is related to olive oil authenticity, which, according to the U.S. Pharmacopeial Convention Food Fraud Database, is the most frequently adulterated food, even if there are clear regulations and specifications related to this product [59]. Other high-priced edible oils (e.g., argan, sesame, and sea buckthorn), most of them used as supplements, are also the subject of counterfeiting. Over the years, in addition to the authentication of edible oils by GC procedure, which is not an easy and practical solution (time-consuming, not appropriate for a large number of samples, and uses toxic solvents), other approaches, such as vibrational or NMR spectroscopy, have been proposed as efficient alternatives, especially in combination with chemometric or AI methods.

In this context, one study that aimed to provide a simple method for discriminating edible oils in rapport with their botanical origin and classifying unknown samples applied principal component analysis (PCA) to several datasets containing one or more parameters (fatty acid profiles, tocopherol values, PCI, and CIELAB parameters) that are currently used in the edible oils industry for their evaluation [60]. Other studies presented the efficiency of a methodology based on various chemometric analyses (PLS, LDA, and SIMCA) and vibrational spectroscopic data for rapid authentication of edible oils [60,61,62,63,64]. Fatty acid profiles, obtained by GC/MS, GC, EI-MS, GC-IMS, or triacylglycerol composition, from supercritical fluid chromatography (SFC) coupled with quadruple time-of-flight mass spectrometry (Q-TOF-MS), in combination with chemometric methods, have also been employed to identify efficient discriminant models [30,65,66,67,68,69].

The involvement of AI tools, especially RF classifiers, for edible oils evaluation proved to be very efficient when applied to fatty acid profiles from GC/MS [30], triacylglycerol profiling obtained by MALDI-TOF mass spectrometry [70], and Raman spectroscopic data [71]. For example, in the case of association with the fatty acid profile, the use of the RF method highlighted the importance of low-abundant fatty acids to the classification, allowing access to more information about the contribution of each variable involved in the classification [30]. To eliminate complex analyses, some authors proposed, for the classification of olive oils (extra virgin, virgin, and refined) or their geographical origin identification, the use of multi-parametric time-domain NMR relaxometry data in combination with ML algorithms. Thus, supervised learning models such as neural networks (NN), logistic regression (LR), naive Bayes, and RF were successfully used for training the datasets, and the obtained results highlighted increased sensitivity and specificity for classifying the olive oil samples using NMR relaxation-based detection (AUC = 0.95) as compared to conventional techniques such as NIR (AUC = 0.84) and UV-Vis (AUC = 0.73) spectroscopies [31]. The comparison between PCA and XGBoost ML methods applied to data resulting from the official analytical methods of the International Olive Council (IOC) showed good results for ML algorithms, both for the cultivars and country of origin classifications [32].

Other AI models such as ANN or CNN have also been utilized in edible oils pattern recognition; thus, ANN in combination with GC analysis was used for vegetable oils classification [72], while CNN was applied for edible oils low-field nuclear magnetic resonance (LF-NMR) data analysis, proving to be an efficient automated approach for edible oils evaluation [73]. A critical review regarding the performances of ANN models in olive oil production, characterization, and authentication applications was published by Gonzalez-Fernandez et al. [74].

As a general overview, very few studies were realized for simple edible oils classification, with most of them carried out in connection with adulteration evaluation purposes; the use of AI tools for the analysis of big data, which are more and more frequently used during the current challenges in authentication studies, proved to be very promising for authentication studies in comparison with the conventional chemometric ones, especially in relation to the supply of more accurate prediction results.

### 3.3. Fruit Juices

The authentication of fruit juices is a complex task due to the wide variability in natural composition comprised by the sugar content, acidity, and volatile compounds, which can vary significantly depending on cultivar, ripeness, climate, soil, and processing methods. Authentication studies are important for determining the characteristics of samples in order to detect if potential adulterants are added, such as water or colorants; if the product is mislabeled due to the replacement of an expensive variety with a cheaper one; or if the declaration of origin is false. In the case of fruit juices, several studies aimed at the identification of the fruit variety of such products, being motivated by fraudulent practices involving the mixture of different fruit juices. While the juice-to-juice adulteration matter is further described in the following section, the classification of fruit juices with respect to the botanical source and origin is addressed herein.

For fruit juice authenticity, it was observed that the composition of sugar and/or organic acid provides information about fruit juice origin [33]. Moreover, stable isotope ratios analysis [75,76], HPLC [77], mass spectrometry [78], elemental fingerprinting [79,80], NIR [34], and fluorescence [81] analytical techniques have been exploited either on their own or in corroboration with statistical tools in order to develop fruit juice authentication models with regard to their varietal and geographical source.

High classification rates were obtained by the authentication models capable of discriminating between laboratory-made fruit juices of apple, pear, peach, grape, sweet cherry, strawberry, and blueberry, constructed on HPLC data and chemometrics (PCA and LDA) [33]. This type of analytical data does not require the use of complex methods such as AI-based techniques for data processing, as the differences between classes are significant. High accuracies can be achieved just with the use of state-of-the-art processing techniques. The ripening stage of mango fruits used in juices was also successfully determined by quantifying glucose, fructose, and sucrose from the PLS-processed MID-FTIR-ATR data [82].

Even though promising results have been achieved through the application of statistical approaches, in recent years, AI techniques have also been employed in juice authentication from a desire to decrease the response time while maintaining or improving model accuracy. In this regard, near-infrared transflactance spectroscopy with a fiber optic probe was used to determine individual sugar content and to identify different varieties of citruses [34]. This time, the PLS statistical method was applied to determine the sugar contents, while ANN-CA was applied to predict citrus variety within less than one minute [34]. IR spectroscopic data of samples containing different concentrations of apple juice were also processed using ANN and yielded satisfactory classification results [83]. The models were improved when a variable selection step was performed by means of genetic algorithms (GA) in order to decrease the training time [83]. Brendel et al. developed high-accuracy models for differentiating between different citrus juices using MS or ion mobility spectrometry (IMS) data and LDA [78]. A low classification performance was obtained by the k-nearest neighbors (kNN) and SVM models. Additionally, low- and mid-level data fusion did not improve the prediction ability as compared to the model constructed on the single data.

For fruit juice varietal discrimination, recent studies have discussed the application of ANN on the results obtained by E-nose and E-tongue for developing highly performing models [84,85] that do not require trained researchers for analytical acquisition or for data processing.

Based on the reported studies, statistical methods were successfully applied for fruit juice label authentication. AI did bring some advantages to fruit juice authentication, which are related to its independence from human input and its more rapid training time.

### 3.4. Dairy Products

Studies in European countries have indicated the importance of dairy product consumption for the supply of essential nutrients for human health [86,87]. In this regard, these types of commodities require quality monitoring, and as a result, there has been an increased interest in developing authentication models for milk, cheese, and other dairy products, as each matrix requires a special focus on its particular weaknesses along the production chain. Two main issues that need to be addressed in milk control are related to (i) the thermal process that milk undergoes and (ii) milk’s animal origin, while for cheese, the main authentication fraud is related to the false declaration of the production area.

Heat treatment is essential for ensuring the bacteriological safety of milk; however, uncontrolled high-temperature heat treatments can lead to the degradation of its nutritional value and the loss of aroma or sensory qualities and can even generate harmful compounds [88]. Because of these, models for the accurate and fast discrimination of thermally treated milk samples have been developed using different analytical techniques (Table 1). For processing the data, either statistical methods or AI tools have been used. No general conclusions could be reached as to which processing method gives the best performance, as few studies have compared the two data processing approaches, while the ones applying both statistical and AI methods reported similar performances.

In this regard, Raman spectroscopy paired with PLS-DA allowed discrimination between raw and pasteurized milk with an accuracy higher than 90% [89]. From our literature review, the best discrimination model with respect to the degree of heat treatment applied to milk was achieved when IR spectroscopy was coupled with random forest (RF) (with an accuracy of 97%), being a slightly more accurate classifier as compared with SVM and kNN (with accuracies above 90%) and a significant better classifier than LDA (whose accuracy was only 84%) [35]. MS was observed to be suitable for assessing heat intensity by identifying differential ions. From this perspective, MALDI-TOF-MS profiling coupled with fourteen ML algorithms was compared to determine the best model for identifying the mild thermal processing of milk samples [90]. The top four algorithms with the best performances were LDA, penalized discriminant analysis (PDA), RF, and SVM, having accuracy scores above 96%.

Identifying the animal origin of milk is important for maintaining the integrity of the dairy industry and to protect consumer health. Depending on the animal origin, the content of the various milk nutrients can vary; for example, sheep milk is, in general, richer in proteins (casein), while goat milk contains more potassium (K) [91,92]. As cow milk production is the largest, the price of this commodity is lower as compared to sheep or goat milk, so authentication of animal origin of milk can prevent fraudulent partial or total substitution practices. In this regard, several analytical techniques have been proposed for the identification of milk types: capillary electrophoresis for the classification of cow and buffalo milk [93] and mass spectrometry for differentiating milk from eight different animal species, namely cow, water buffalo, wild yak, goat, sheep, donkey, horse, and camel [94]. These methods do not require statistical or AI treatment of data.

Recently, fast and reliable spectroscopic techniques have been extensively applied in dairy authentication, resulting in the need of employing statistical methods to process the spectroscopic data. In this regard, FTIR spectroscopy was successfully used to discriminate goat from sheep milk [95], buffalo from goat milk [96], and cow from goat milk [97]. Recently, laser-induced breakdown spectroscopy (LIBS) assisted by ML was proposed for the identification of milk animal origin (cow, goat, and sheep) of 1296 raw liquid milk samples, obtaining an LR model with an accuracy of 92.8% [98]. As a general tendency, when spectroscopic data were used to construct milk authentication models, statistical methods and AI tools proved to have nearly the same efficiency.

The second most consumed dairy product is cheese. Its composition depends on the animal and geographical origin, the production and condition technology, and ripening time, all of which give sensorial and nutritional differences among cheeses in terms of taste, acidity, casein, proteins, calcium, and phosphorus content. Specific European certifications are given to the officially recognized cheese varieties that have specific production areas, and include Protected Designation of Origin (PDO), Protected Geographical Indication (PGI), and Traditional Specialty Guaranteed (TSG) [99]. The certified products present a higher market value than other similar dairy products and are more susceptible to fraud. Therefore, developing authentication and traceability models is necessary to protect their geographical indications and designations of origin [100]. Such reported classification models have been developed using different analytical approaches in corroboration with statistical or AI methods. As there are a great number of studies focused either only on statistical tools or only on AI tools, and very few studies contain a proper comparison between these two [4], it is difficult to assess whether AI provides any significant improvement to the authentication model performances.

From this perspective, the origin and authenticity of PDO Polish cheese, Oscypek, was assessed based on its volatile profile, determined by using a solid-phase microextraction–mass spectrometry method (SPMEMS) in corroboration with PCA, LDA, SIMCA, and SVM. For all statistical approaches, the model performances were comparable [4]. The potential of the volatile profile in the characterization and discrimination of three Italian pecorino cheeses was assessed using GC-MS combined with HS-SPME and two supervised multivariate statistical approaches [101], which provided an overall classification accuracy (in external validation) of 87.5%.

The mineral composition of Brazilian artisanal cheese was determined, and cheese classification models with high accuracies were obtained by inductively coupled plasma–optical emission spectrometer (ICP-OES) and ML algorithms [36]. RF and SVM were the most accurate models for differentiating between ripened and non-ripened cheeses, while for the classification of the production region, all the algorithms presented excellent performance scores [36]. The free volatile carboxylic acids method (FVCAs) was employed to describe 10 different Swiss cheese varieties, and by using ML techniques, 90% of the test data was correctly classified according to the cheese type [102]. The chemical parameters obtained by electrophoresis and chromatography and ANN allowed for an efficient and accurate prediction of the production area of the Ossolano cheese [103]. Moreover, the use of GA optimized the input space, leading to superior recognition accuracies and significantly decreased learning time [103].

### 3.5. Meat

Consumers concern regarding the authenticity of meat products along the processing chain demanded the development of authentication models based on analytical techniques for quality assessment and composition determination of meat and its derivative products. Besides the reported studies aiming at adulteration identification and quantification in meat, a topic that is discussed in the following sections, the classification models proposed in the literature are mostly related to the discrimination of (i) fresh and thawed meat, (ii) species differentiation, (iii) meat from different parts of the animal, (iv) meat samples from distinct geographical origins, or (v) rearing systems (i.e., yard or industrial).

Traditional analytical techniques used for differentiating fresh and thawed meat include enzymatic-based methods applied to chicken meat [104], DNA [105], spectroscopic techniques for beef [106] or fish [107] freshness, or MS [37]. Most of these studies obtained a high efficiency in discriminating the meat types using statistical methods, whereas the others did not require the use of any data processing strategy. The differentiation of species has been addressed in many studies involving different techniques, from the traditional low-detection and reliable methods such as PCR and real-time PCR techniques [108] to the fast and non-destructive spectroscopic techniques [109,110].

For differentiation among different chicken parts from minced or non-minced samples, NIR spectrometry paired with distinct supervised methods such as LDA, RF, and SVM was applied. The reported performances of the classification models did not illustrate the advantage of applying one algorithm over another [110].

In the recent study by Cristea et al. [38], the application of ANN in corroboration with isotope and elemental concentrations was shown to be a reliable approach for identifying the geographical origin of pork meat samples as well as the rearing system. In this case, the AI-based models outperformed the ones developed using LDA.

The possibility of predicting with high precision the geographical origin or growing system quality factors of meat based on mass spectrometry techniques and supervised statistical methods was also illustrated in the studies of Zhao et al. [111].

Based on the performed literature review, it can be highlighted that in the case of meat, there are numerous studies that have aimed to detect mixtures of distinct meat types or other adulteration issues, as is highlighted in the following sections.

## 4. Application of AI in Food Adulteration Detection

**Figure 3:**
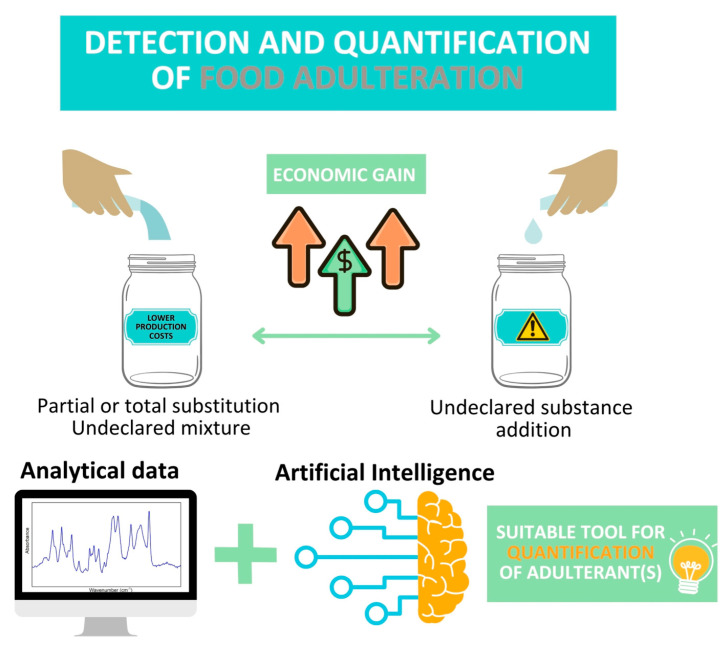
The main advantage provided by AI over statistical methods in terms of adulteration percentage estimation.

A step forward regarding AI application in food science was made through its engagement in fraud control to detect partial or total substitution of certain ingredient(s) or an undeclared mixture of varieties (Figure 3). This tendency appeared because the development of reliable models for adulterant detection and quantification has benefited from increased attention in recent years. These models were developed based on various experimental data obtained through traditional analytical tools (i.e., MS-based techniques) and faster and easier-to-use spectroscopies (Table 2). As was previously stated, the optimal choice of data processing treatment depends on several factors: the investigated food matrix, the adulteration issue, the analytical method used, and sample distribution. For this reason, each matrix is discussed separately.

### 4.1. Honey

For honey, which is a very complex matrix, many studies related to its adulteration have been conducted over the years. A direct addition of different substances or low-cost varieties in honey can be noted as a general practice found on the market. The indirect adulteration by the over-feeding of bees with sucrose solutions or crystalline industrial sugar is also a significant concern, especially because this type of adulteration is very difficult to detect.

Thus, the main tendency in honey adulteration is related to the direct addition of sweeteners such as glucose, fructose, sucrose, maltose, corn, cane, beet, rice, barley malt, inverted sugar syrups, or even colorants such as ammonia or sulfite ammonia caramel. Moreover, from an economic point of view, the mixture of high-value honey types (e.g., manuka) with more accessible and low-cost honey varieties (e.g., colza, sunflower, etc.) also has a significant impact on the honey industry. Therefore, many studies have been conducted for the identification of adulterated samples, some of them using techniques such as NMR, vibrational, UV-Vis, or fluorescence spectroscopies that allow a more rapid evaluation of samples. In many cases, the large datasets obtained were analyzed by various chemometric methods such as LDA, PLS, and SIMCA, allowing the discrimination of adulterated honey with high efficiency. A comprehensive review of honey adulteration detection by various methodological procedures was published by Brar et al. [126].

In the last few years, several spectroscopic techniques (NMR, Raman, MIR, or Vis-NIR) have been used in combination with supervised ML methods for the identification of honey adulteration by either the direct addition of sweeteners or by mixture creation with cheaper honey. Thus, the recognition of sugar adulterants in honey was made possible by combining MIR analysis with a 1D-CNN model when acacia honey was adulterated with corn syrup [112]; ^1^H-NMR data were also analyzed by LR, DL NN, or light gradient boosting classifiers for detecting brown rice, corn, or jaggery syrups in adulterated rapeseed honey samples [114]. Raman spectroscopy in association with CNN also proved its efficiency in identifying the adulteration of common lychee honey with high-fructose corn, rice, maltose, or blended syrups [113]. An analysis of the Raman spectral data by convolutional or probabilistic neural networks (CNN or PNNs) or even SVM models allows adulteration detection of Suichang native honey with maltose syrup [127].

ML algorithms have also been involved in identifying a more subtle adulteration that is obtained by mixing two types of honey (Table 2). Thus, using Vis-NIR, Raman, or ATR-FTIR spectroscopy in combination with various ML models, it has been shown that these approaches are efficient for detecting the addition of various concentrations of low-cost honey [9,115,119]. In this context, the identification and quantification of honey-based adulterants in two types of honey, orange blossom and sunflower, was performed through Vis-NIR and SVM or RF, and 100% accuracy was reported for both models, but no details were given about the nature of the honey-based adulterants [119]. Various concentrations of acacia/colza honey mixtures were detected through the association of Raman spectroscopy and kNN with an 88.6% accuracy [115]. Also, ML approaches applied to the ATR-FTIR datasets of acacia/colza or linden/sunflower honey mixtures highlighted the potential for differentiating these mixtures from the appropriate unadulterated samples, with an accuracy of 94.4% and 90.7%, respectively, when using the linear discriminant model [9].

Based on the reported results, it has been proven that the application of AI for food fraud control enables the detection of subtle adulteration types (like those obtained through the undeclared mixture of honey) and also the estimation of its degree.

### 4.2. Oils

Possessing a large composition range due to botanical origin, geographic and climatic environment, seed quality, extraction and refining processes, or storage conditions, edible oils are essentially composed of triacylglycerols (95–98%) and various mixtures of minor constituents (2–5%) [128]. Regarding oil adulteration, two practices can be considered significant: the mixing of cold-pressed oils with refined ones and the substitution of some valuable oils with more accessible and cheaper oils [129].

Thus, the use of chemometric methods (mainly PCA, (S)LDA, or PLS) in combination with various analytical techniques allowed the development of reliable tools for oils’ investigation. The adulteration of oils can be detected either by considering the fatty acid profiles obtained from GC techniques or other spectroscopic techniques [30,66,129]. Some methodologies based on electronic nose analysis [130] or low-frequency dielectric spectroscopy [131] combine both chemometrics and ANN techniques for oils’ evaluation and prediction of the adulteration degree.

However, in many food adulteration cases, the main issue is strongly correlated with the lack of information about the type of involved adulterants, and simultaneously, the control interest is mainly related to the identification of adulterated samples. In this context, there are many discussions about the efficiency of chemometrics tools based on binary or multiclass classification methods for authentication/adulteration purposes since the adulterant is unknown, or there are many distinct adulterants [132]. The proposed solutions involve the use of one-class classification models, either pure one-class or modified classifiers, or the involvement of the RF algorithm for one-class problems in combination with the artificial generation of outliers for model building [132]. A study involving Raman data and the fatty acid profiles (GC) of several commercial edible oils (i.e., avocado, canola, coconut, liquid coconut, corn, grapeseed, olive, peanut, soybean, and sunflower) highlighted the performance of ML-based algorithms (PCA with RF) in comparison with the standard PCA model for sample classification based on Raman data, while LNR was the most efficient model for predicting both adulteration cases: avocado oil by canola oil and olive oil by soybean oil [71].

Considering the identification of various adulterated oil samples with the help of other AI techniques (either traditional or DL methods), it is worth mentioning that the involved methodologies make use of different analytical techniques, i.e., IR, Raman, or fluorescence spectroscopy and even chromatography (either GC or HPLC). An example involving Raman spectroscopy in association with an ML-based model evidenced the efficiency of an ensemble–subspace kNN model for identifying the adulteration of sea buckthorn oil by sunflower and pumpkin oils [7]. The use of SVM algorithms revealed excellent results for the adulteration of EVOO with rapeseed and corn oils when applied to the chromatographic data of pigments [116].

A complex study based on the fatty acids profiles obtained by GC-FID techniques, involving ten edible oil types, demonstrated that DL allows the discrimination of unknown oils types more efficiently than chemometric methods [117]. This study also reported the lack of chemometric models’ efficiency if the products’ complexity is increased.

Thus, for the oil matrix, these studies clearly confirm that the effectiveness of AI in comparison to statistical methods directly increases with the adulteration subtlety.

### 4.3. Fruit Juices

Based on the reported studies presented in Table 2, fruit juice adulteration can occur from undeclared substance additions (i.e., water and different sweeteners like glucose, fructose, corn syrup, organic acids, or fruit byproducts) to more subtle adulterations like those performed through the mixture of a certain fruit juice (i.e., orange juice) with a cheaper variety (i.e., grapefruit). In this regard, statistical methods have been successfully employed to develop models that sense the presence of other added substances in the composition of fruit juices, such as water, sugars, and organic acids [133,134].

Regarding the addition of different substances, a study by Lyu et al. aimed to develop a new approach combining LC-MS-based metabolomics and to distinguish between authentic and adulterated lemon juices obtained by the addition of flavonoid markers. PCA and PLS-DA were applied to observe relevant cluster patterns, while for the prediction of the adulteration, five ML methods were employed, from which SVM led to the most accurate predictions [118].

A DL-based approach for juice quality analysis was reported by Malek et al., who proposed a three-layer CNN for analyzing the sugar concentration in adulterated orange juice [120]. The NIR features extracted from the 1D-CNN model significantly improved the performance parameters when compared to those obtained by AlHichri et al., who used the state-of-the-art chemometric regression methods on the same sample set [121].

For the detection of pomegranate juice substitution by cheaper apple or grape juice, Raman spectroscopy in conjunction with PLS and SVR was successfully used with similar performances [5]. The NIR data processed through LDA for detecting juice-to-juice adulteration proved to be more effective than the SVM model. In contrast, for the quantification of adulteration, the best performance was obtained by applying SVR, emphasizing the efficiency in quantifying subtle adulterations [6].

Based on the reported data, we can state that data processed by AI tools for developing detection and quantification models for fruit juices presented a higher efficiency than the models based on statistical methods. As compared to the authentication of fruit juices (discussed in the previous section), where no clear difference between the performances of these data processing categories could be observed, in the case of adulteration detection, AI proved to be the most efficient and accurate. This might be linked to the fact that fruit adulteration, especially in small quantities, is more difficult to detect.

### 4.4. Dairy Products

In the case of dairy products, the partial or complete replacement of the original product with more easily available and cheaper substances/products is the most common procedure performed by defrauders. The addition of other compounds in the composition of dairy products to improve flavor or properties also represents an authentication issue [135]. In this regard, more expensive kinds of milk were reported to be adulterated with different types of cheaper milk, or other compounds, such as whey, neutralizing agents to mask acidity, melamine, salt, or sugar, were added to mask extra water [136]. Other fraudulent means consist of the excessive addition of water or the addition of non-milk fat/oil, which results in a decrease in the nutritional quality of the dairy products.

Cheese whey addition to milk was until recently detected using HPLC, a method that does not require any statistical treatment for the determination of the adulteration degree but has as its main disadvantages high costs and complexity of the analytical method. In recent years, the faster, reliable FT-NIR spectroscopy method together with AI tools, such as classification and regression tree (CART) and multilayer perceptron, has been proven capable of detecting the addition of cheese whey to milk with high accuracy [122], thus mitigating the analytical complexity and associated costs through the enhanced capabilities of AI.

The detection of melamine in dairy products was assessed through a model developed on NIR/MIR spectroscopies in conjunction with statistical methods (PLS) or ANN and LS-SVM. A comparison between model performances pointed out the better performance of AI-based models [123]. Moreover, the models constructed by Neto et al. based on FT-IR spectroscopy for the detection of sucrose, starch, bicarbonate, peroxide, and formaldehyde addition led to better accuracies when AI tools were applied instead of supervised statistical methods (e.g., PLS) [137]. In another study, milk adulteration was detected with 100% accuracy by combining differential scanning calorimetry with ML tools (gradient boosting machine (GBM) and multilayer perceptron) [138].

Ayari et al. successfully detected sunflower oil and cow body fat mixed with pure cow ghee using an E-nose system in corroboration with ANN [139]. In a study that aimed to distinguish and quantify non-dairy cream present as an adulterant in milk fat cream, both OPLS-DA and ML algorithms were applied to REIMS lipid fingerprints. The chemometric method was limited in identifying or quantitatively analyzing traces of non-dairy cream adulteration. Thus, a refined classification and quantification with an accuracy above 98% was achieved when ML models (DT, SVM, and ANN) were employed [124].

AI allowed the development of effective approaches when sensors were involved in adulteration detection. Thus, Tripathy et al. developed and evaluated a paper-based, scalable milk pH sensor [140]. The sensor used the RGB values of the colored fiber and AI algorithms (SVM and kNN) to discriminate pure milk and to quantify the pH value of a milk sample (with accuracy over 98%) to prevent tamper-proof or spoiled milk adulteration. Another low-cost, portable AI-based sensor was applied to detect milk adulterants using the UV-Vis spectra of the analyzed samples [141]. The developed AI-based model was capable of differentiating between five adulterants, with accuracy scores between 88% and 92%.

Based on the reported results from the literature, the efficiency of AI for dairy authentication has proven to be more suitable for fraud detection than statistical tools.

### 4.5. Meat

The development of detection tools for meat products adulterated either with cheaper or spoiled meat, animal offal, or non-meat materials has high practical importance. The traditional detection methods such as chromatography and DNA-based techniques [142,143]; protein markers for discrimination of meat species in raw beef, pork, and poultry and their mixtures [144]; and even spectroscopic techniques such as UV–Vis [145] and Raman [146] have been successfully applied for adulteration detection and quantification of meat in conjunction with supervised methods.

As previously highlighted, studies usually choose supervised statistical methods for model development to discriminate between adulterated and authentic samples. A different approach was used by Pu et al. when studying 582 samples of beef meat adulterated with other animals’ meat by using MALDI-TOF MS and XGBoost, developing a model with an accuracy of 97% [125].

In the field of meat authentication, HSI proved to be an effective analytical tool that attracted many research groups to pay a lot of attention to the development of such types of detection approaches. Taking all of this into consideration, this subject is described in detail in the following section dedicated to image processing.

## 5. Image Processing

**Figure 4:**
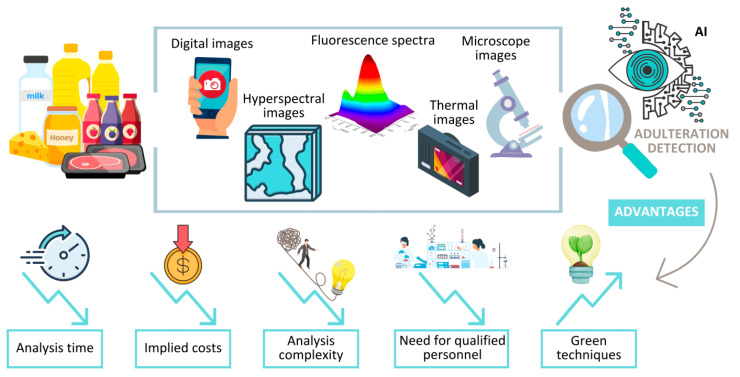
A new road provided by AI for food control. Images as a data source for food fraud detection.

During the last few years, new approaches dedicated to food fraud detection, based on image processing with the help of AI, have revealed amazing results in terms of accuracy and ease of use. As a function of the matrix type, adulteration issues, equipment types, and expertise, the association between the method for image acquisition and the AI treatment is different among the reported studies (Table 3). Moreover, this field is in an emerging phase and has an amazing potential to develop new, effective, easy-to-use, portable devices for food control (Figure 4). As the reported results are matrix-oriented, a screening of the latest results reported for each discussed food item is further presented.

### 5.1. Honey

Even though several analytical techniques were successfully applied to determine the honey botanical source (see Section 3), the traditional and certified method remains melissopalynology, a technique referring to the study and examination of pollen grains found in honey using light microscopy. However, as the melissopalynological method is laborious, demands expertise from specialized individuals, and entails a meticulous counting process, it makes botanical source identification very challenging [156]. Against this background, DL has unlocked new possibilities for the development of tools able to automatically and rapidly identify pollen grains, to recognize their type, and to determine, based on a microscope image, the botanical origin of the honey.

In the field of melissopalynology, several CNN models have been proposed for the classification of pollen grains with respect to their botanical source, for example, the ones proposed in the work of Sevillano and Aznarte [157], but only a few have focused on the automation of pollen grain identification in images of honey analyzed through optical microscopy [158].

AI-based techniques have also been successfully applied for the detection of subtle adulterations in honey based on infrared images. The study of Izquierdo et al. investigated the potential of applying DL for the detection and quantification of rice syrup in honey in concentrations between 1% and 8% using infrared thermography [147]. For this purpose, the authors proposed the use of CNN to extract patterns from the thermographic images recorded during the cooling process of adulterated and pure honey belonging to two botanical origins, namely acacia and lemon. The ability of the proposed model to identify honey adulteration with rice syrup independently of the botanical origin corresponded to a 95% accuracy score on the test set, while a 92% accuracy was obtained for predicting the concentration of the adulterant in acacia or lemon samples during the test phase. The lowest performance was recorded in the case of samples consisting of 1% rice syrup; namely, a true-positive rate of 81% was obtained for this counterfeit honey class during testing.

### 5.2. Oils

The application of DL for differentiating EVOO, virgin olive oil (VOO), and lampante olive oil (LOO) samples based on the images acquired through GC-IMS was investigated by Vega-Márquez et al. [150]. For this purpose, a dataset of 701 images was employed for the development of a CNN model able to simultaneously discriminate among EVOO, VOO, and LOO samples with an accuracy of 82.8% over an independent test set. The work was reported as a step forward in developing a fast and cost-efficient tool for olive oil classification with respect to their previous study [159], which illustrated the application of feed-forward ANN starting from the same dataset but involving the manual extraction of features from the recorded images.

Another application of DL in the field of oil authentication is represented by the work [149], who reported for the first time in the literature the study of the thermal profile of oils during the cooling process with the aim of identifying and quantifying the adulteration of EVOO with refined olive oil, olive pomace oil, and sunflower oil. Their motivation was linked to the fact that the composition of triacylglycerols in oil samples influences their thermal characteristics. A thermographic camera was used to capture the thermal evolution from 45 °C to 25 °C of both pure and adulterated samples, and the resulting images were used for constructing distinct CNN models for classifying EVOO samples and for determination of the adulterant concentration. The reported accuracy ranged between 97% and 100%, proving the efficiency of the proposed solution.

In their study, Pradana-Lopez et al. [148] highlighted the efficiency of applying CNN for classifying distinct EVOO and for the semi-quantification of sunflower and corn oil adulterants in EVOO based on images acquired through optical microscopy. The baseline of their work corresponded to the idea that each oil possesses a unique rheological property, which was able to be learned by the CNN model by examining images that captured the expansion of oil droplets over a 30 min period. The dataset comprises a total of more than 302,000 images of authentic and adulterated oil droplets. The optimized CNN model led to an impressive accuracy score of 96% in predicting the authenticity or the adulteration rate (between 2.5% and 10%) of the EVOO despite the simplicity of the data as compared to other experimental data used in the studies presented in Section 4.

CNN models were also successfully applied to extract features from the 3D fluorescence spectra of several types of vegetable oils to detect and quantify adulterants in sesame oil samples through the subsequent application of SVM and PLSR, respectively [10]. Through this approach, the SVM model constructed on the basis of the extracted features allowed the correct detection of adulterated samples in the test set as well as the identification of the adulterant type (e.g., rapeseed oil combined with sesame oil essence) with 100% accuracy. However, when the input data corresponded to the emission spectra at an optimal excitation wavelength, the performance of the SVM model decreased, i.e., 91% of the samples were correctly predicted for the same task. Lastly, through the application of DL for spectral feature extraction, PLSR models were successfully developed for the quantification of sesame oil essence in counterfeit samples. This approach led to RMSEP values between 0.99% and 2.20%, which proved the reliability of the proposed solution.

### 5.3. Dairy Products

AI has also been applied for the development of new approaches based on image processing in the field of dairy product quality control. In this regard, Visconti et al. [151] proposed the application of digital imaging for the development of a rapid and cost-effective tool to detect adulteration in grated cheese through the addition of additives above the approved limit (i.e., cellulose, silicon dioxide, etc.) or other volume enhancement substances like wheat flour, wheat semolina, or sawdust. For constructing classification models, mean color histograms were computed based on the acquired digital images. Several statistical and ML methods were used for this purpose, namely SVM, random trees, DT, LR, kNN, and PLS-DA. Based on this approach, accuracy scores between 50% and 81.7% were obtained by the constructed models, and the highest prediction performance corresponded to the SVM model, which was able to identify pure samples or adulterated samples with a precision greater than 75%.

AI has also facilitated the development of an efficient, rapid, and non-invasive tool capable of automatically and precisely determining the degree of ripening of pecorino cheese based on images captured by a photo camera [11]. The study of Loddo et al. investigated the application of CNN and traditional ML techniques (i.e., SVM, kNN, RF, DT, and ANN) using both handcrafted and deep features extracted from the acquired images [11]. The obtained classification results indicated that the association between CNN as a deep feature extractor and SVM as a supervised classification technique leads to the best performance in predicting the degree of ripening (i.e., 18, 22, 24, or 30 days) of pecorino cheese. Nonetheless, the study highlighted a new possibility for dairy product control using an accessible means of capturing specific discrimination characteristics, namely a photo camera.

### 5.4. Meat

In the field of meat quality and safety assessment, hyperspectral imaging (HSI) has become a promising and widely applied technology that is able to provide both spectral and spatial information about the investigated samples in a rapid and non-destructive manner [160]. HSI systems have been successfully applied for predicting numerous quality parameters in meat samples, such as pH value [161,162,163], tenderness [163,164], color [162], intramuscular fat [161], or marbling [165,166]. Furthermore, HSI has proved to be a powerful tool for the identification of minced meat adulterated with other meat types [167,168] and with other substances [169].

Due to the nature of HSI data, the application of statistical or conventional ML methods like SVM or kNN for constructing meat recognition models is commonly conducted after a data dimensionality reduction step [24]. In this regard, several approaches have been investigated, for example, the averaging of the pixel-wise spectra corresponding to the region of interest (ROI) [170], the application of PCA [171], and spectral angle mapping [172], among others. Another solution for overcoming this limitation and enabling hyperspectral data processing through such statistical or learning-based methods is treating each pixel-wise spectrum as an independent sample [152].

Even though promising results have been achieved through this type of approach, recent studies highlight the potential of CNN for the automatic extraction of features from hyperspectral data, which has been shown to be more efficient in meat quality and authenticity studies. For example, in the study of Al-Sarayreh et al. [152], the application of CNN for the extraction of spectral and spatial features allowed the identification of the type of red-meat muscle, irrespective of the status of meat (i.e., fresh, frozen, thawed, packed, or unpacked), with an accuracy of 94%. This approach proved to be more efficient than SVM modeling for the same purpose based on handcrafted features. These results are in good agreement with the study of Ayaz et al. [155], which emphasized the higher ability of CNN as compared to SVM or kNN models in differentiating three minced meat types (i.e., beef, mutton, and chicken). The robustness and time-efficiency advantages of 3D-CNN modeling for processing HSI data of meat samples were also highlighted by Al-Sarayreh et al. [154].

Nonetheless, ML has been successfully applied in conjunction with RGB color imaging to detect and quantify plant- and animal-based adulterations in minced meat [153]. A perfect discrimination between pure and adulterated samples was achieved through the proposed approach, whereas performances corresponding to up to 76.1% accuracies and up to 98% r-values were obtained for identifying the type of adulterant and quantifying it.

Based on the reviewed studies, the employment of AI for image processing can be regarded as a step forward for meat control, allowing a fast and accurate assessment of both authentication and adulteration.

While AI-based methods have shown significant potential in food authentication and quality control studies, several limitations must be acknowledged. Model generalization remains a key challenge, as AI models often perform well on specific datasets but may struggle when applied to new data from different sources, regions, or production conditions. In order to reach a clear conclusion regarding the advantages of using AI as opposed to statistical methods, more studies comparing the two tools are required. Another limitation of AI tools is the lack of standardization in data acquisition protocols, which can significantly affect model performance and reproducibility across laboratories or industries.

## 6. Conclusions

Based on the reported studies, AI-based approaches have been increasingly applied for the differentiation of distinct food commodities with respect to numerous label attributes, from geographical or botanical origins to fabrication technologies. The involvement of learning-based techniques in the field of food authentication can be regarded as a logical progression toward improving the performance of recognition models. However, a clear conclusion regarding the advantages of these techniques over statistical methods has not yet been reached, as several studies employing both ML and statistical methods showed an insignificant performance superiority of the first category, while others showed a slight decrease in the prediction ability. As a future perspective, more studies involving comparisons of these methods performed on the same dataset, highlighting the advantages and disadvantages, are needed.

For the adulteration detection, it was highlighted that AI is more effective as compared to chemometrics for the detection of subtle food frauds, like those performed through the undeclared mixture of different varieties belonging to the same matrix but having significantly different commercial values (i.e., manuka honey and a common variety).

The use of images in the field of food authentication has uncovered new possibilities for a fast assessment of several food matrices, including honey, meat, oil, and dairy products. In this regard, for the development of food recognition models, DL has shown great potential, especially in deep feature extraction, eliminating the need for specialized personnel while offering high efficiency and facilitating a rapid and practical analysis for real-time and scalable deployment.

Based on these considerations, new perspectives are foreseen in the development of reliable, easy-to-use, portable tools based on the association between images and DL. The key advantages of the application of AI-based approaches in the field of food quality and control are related to their feature learning and higher generalization capability as opposed to conventional statistical methods, their ability to provide more accurate predictions, and, more importantly, to decrease the dependency on expert knowledge or human involvement.

Additionally, the increase in the developed authentication tools, despite the obvious positive effects, needs to be approached with caution and, at least in the near future, only used as a screening method that can substantially increase the number of samples that can be easily controlled. All suspected samples need to be investigated through the acknowledged methods for a final verdict. This is because of the natural variability of such complex matrices as food commodities that require a spatial and temporal representative learning dataset, which is not trivial to achieve. Nevertheless, the development of screening tools is a huge step forward that can decrease the dependency on expert knowledge or human involvement.

## Figures and Tables

**Table 1:** Selection of research articles related to food authentication with respect to distinct label attributes through the application of AI or statistical methods.

Product	Aim	Experimental Data	Processing Method(s)	Performance	Ref.
Honey	Classifying six varieties of Chinese honey (linden, sunflower, vetch, rape, acacia, and jujube)	IRMS, ICP-MS	RF, SVM, LDA, CART	The prediction accuracy of the RF model (96.5%) was better than SVM (91.5%), LDA (88.8%), and CART (82.1%)	[26]
	Discriminating the botanical origin of Anatolian honey samples	ATR-FTIR	PCA, HC	Sample discrimination was achieved successfully	[27]
	Botanical origin prediction of honey samples	NIR	PLS-DA, SVM	PLS-DA: around 80% accuracy, SVM: above 90% accuracy for honey classification	[28]
	Honey authenticity control with respect to its geographical and botanical origin	Raman spectroscopy	SIMCA, SVM	SIMCA model provided a better classification of honeys	[29]
Oil	Authenticity detection of five edible oils	GC-MS	PCA, HCA, RF	RF correctly classified the five types of edible oils	[30]
	Classifying olive oils samples and indicating the origin regions	multi-parametric time-domain NMR relaxometry	kNN, LR, NB, NN, RF	Classification of olive oils: AUC = 0.95; tracing the regions of origin: mean AUC = 0.71	[31]
	Development of classification models capable of identifying cultivar origin (Greek or Italian)	GC-MS	XGboost	Sensitivity values for Coratina, Favolosa, Koroneiki, and Lianolia were 0.78, 0.67, 0.71, 0.93, and 1, respectively Specificity values were 0.93, 0.91, 0.95, 1, and 0.98, respectively	[32]
Fruit juices	Discrimination between apple, pear, peach, grape, sweet cherry, strawberry, and blueberry fruit juices	HPLC	PCA, LDA	Discrimination based on sugar content: LDA: 98% CV accuracy; based on organic acid content: above 94% CV accuracy; based on both: 100% CV accuracy	[33]
	Assessment of the origin of citrus fruits	fiber optic NIR spectroscopy	PLS, ANN, GA, CA	ANN and cluster analysis showed great classification power according to the variety and origin, with an R2 value greater than 0.996	[34]
Milk	Discriminating the degree of heat treatment applied to milk	FTIR spectroscopy	PCA, kNN, SVM, RF, LDA	Model accuracies: 0.97 RF; above 0.9 SVM, kNN; and 0.84 LDA	[35]
Cheese	Classifying the Brazilian artisanal cheese (BAC) according to the type and producing region	ICP-OES	ANN, kNN, RF, SVM, LVQ	For the cheese type classification, 0.82 accuracy obtained for the RF and SVM model; for production region discrimination, all classifiers obtained perfect accuracy	[36]
Meat	Fresh and frozen–thawed beef muscle differentiation	REIMS	PCA−LDA, OPLS-DA	The discrimination of fresh and frozen−thawed meat was achieved in real-time in an above 92% accuracy	[37]
	Geographical origin, and animal diet differentiation	IRMS, ICPMS	LDA, ANN	assessment of the geographical origin of tenderloin meat samples: LDA 91.4% accuracy; ANN above 94%; feeding regime differentiation: ANN above 97% accuracy	[38]

**Table 2:** Summary of the employment of AI and statistical methods for identifying or quantifying adulterants in food products.

Product	Aim	Experimental Data	Processing Method(s)	Performance	Ref.
Honey	Identification of sugar addition in honey	MIR	PLS-DA, LS-SVM, CNN	Overall improved average accuracy of the CNN model (97%), over LS-SVM (91%), and PLS-DA (79%)	[112]
	Identification and quantification of honey samples adulterated with high-fructose corn, rice, maltose, and blended syrup	Raman spectroscopy	PLS-DA, PCA-LDA, kNN, CNN	CNN led to a better performance compared with chemometrics (classification by adulteration concentration with a 97% accuracy and a 94.79% accuracy for simultaneously detecting honey adulterated with any type of syrup)	[113]
	Adulteration detection of three major sugar adulterants: brown rice, corn, and jaggery syrup	NMR	LR, DNN, LGBN	99.8%, 99.3%, and 98.7% accuracies for the LR, DNN, and LGBM classifiers, respectively	[114]
	Recognition and quantitative mixture detection	IR or Raman spectroscopy	PCA, PLS-DA, SVM	The acacia–colza mixture detection model allowed an accuracy of 88.6% (kNN); the mixture of colza–acacia obtained an accuracy of 94.4% (LDA); the linden–sunflower honey blend obtained a 90.7% (LDA) accuracy	[9,115]
Oil	Detection and quantification of several edible oil adulterated with sunflower oil	Raman spectroscopy	ML algorithms	Best oil adulteration model accuracy of 88.9% on the kNN model	[7]
	Adulteration identification of extra virgin olive oil (EVOO) mixed with rapeseed and corn oil	HPLC	SVM	Identification and classification of different types of edible oils model had an overall accuracy of 94.44%; SVM model can achieve accurate classification of oil binary blends with a 1% adulteration level	[116]
	Oil fatty acid composition determination and mixture adulteration detection	GC-FID	GMM	The supervised DL model could predict a purity between 91 and 99.5%	[117]
Fruit Juice	To distinguish between authentic and adulterated lemon juices	HPLC/UV–Vis /MS, UPLC-QTOF/MS methods	PCA, LDA, PLS-DA, SVM, RF, NB, LR	LDA: 66.7%, LR 93%, NB: 83%, RF: 84%, and SVM: 96.7% on the CV set (SVM and RF: 100% accuracy for both the training and testing set)	[118]
	Detection and quantification of juice-to-juice adulteration (apple, pineapple, and orange juices adulterated with grape juice)	FTIR	LDA, SVM, RF	Detection of adulteration with good results for all tested methods (accuracies above 97%)	[119]
	Determination of the concentration of saccharose in orange juice samples	NIR	1D and 2D CNN	The PLSR method achieved a better result (NMSE: 0.1626) compared to GPR and SVR; 1D-CNN model NMRSE value of 0.1569	[120,121]
Milk	Detection and quantification of cheese whey adulteration in milk	FTIR	CART, MPNN	Best CART model obtained a high performance with an accuracy of 0.962 and precision, sensitivity, and specificity of 0.965, 0.943, and 0.975	[122]
	Melamine detection in complex dairy matrixes (infant formula, milk powder, and liquid milk)	FTIR	Poly-PLS, ANN, LS-SVM	Limit of detection below 1 ppm could be reached with a multivariate algorithm; the Poly-PLS method was only effective for low concentrations of melamine in milk samples	[123]
Fat Cream	Detection of non-dairy cream in milk fat cream adulteration	REIMS	PCA, OPLS-DA, NN, DT, SVM	OPLS-DA limited in accurately determining or quantitatively analyzing traces of non-dairy cream adulteration; ML algorithms obtained accuracies above 99.0%	[124]
Meat	Detection of beef adulterated with chicken, duck, or pork	MALDI-TOFMS	PLS-DA, XGBoost	Reliable and robust XGBoost classification models with a mean accuracy of 97.4%	[125]

**Table 3:** Overview on the application of AI for image processing in the field of food adulteration detection.

Product	Aim	Experimental Data	AI Method(s)	Performance	Ref.
Honey	Detect commonly elusive rice syrup in honey in concentrations as low as 1% in weight as well as quantify it	Infrared images from a thermographic camera	CNN	95% accuracy for adulteration detection (testing); 92% accuracy for quantification (testing)	[147]
Oil	EVOOs classification, detection, and quantification of adulterated samples for each individual EVOO; a global version of the previous models combining all EVOOs into a single quantifying CNN	Images from optical microscope	CNN	98.3% accuracy on test set 96.8% accuracy on test set 96.7% accuracy on test set	[148]
	Identification and quantification of counterfeit sesame oil	3D fluorescence spectrum	CNN (feature extraction), SVM (classification), PLS (quantification)	100% accuracy (SVM); RMSEP between 0.99% and 2.20% (PLSR) on test sets	[10]
	Identification and quantification of adulterated EVOO containing refined olive oil, olive pomace oil, or sunflower oil	Thermographic images	CNN	97–100% accuracy score on test sets	[149]
	Discriminate among EVOO, VOO, and LOO samples	Images acquired through GC-IMS	CNN	82.8% accuracy on an independent test set	[150]
Cheese	Cheese-ripening monitoring	Images acquired by a photo camera	CNN, SVM, kNN, RF, DT, ANN	98% accuracy by associating CNN (feature extraction) and SVM (classification)	[11]
	Adulteration identification in grated cheese with higher levels of additives	Digital images	SVM, RF, LR, DT, kNN	81.7% accuracy score (SVM)	[151]
Meat	Detecting adulteration in red-meat products	Line-scanning images of lamb, beef, or pork muscles (HSI)	SVM, CNNs	94.44% accuracy (CNN)	[152]
	Detection of plant and animal adulterants in minced meat	RGB color imaging	CV, SVM	100% accuracy in detecting meat adulteration; 76.1% accuracy in identifying the type of adulteration; 98% r-value for quantifying it	[153]
	Red-meat classification (i.e., lamb, beef, and pork)	HSI	3D-CNN	Overall accuracy of 96.9% and 97.1% for NIR and Vis snapshot HSI, respectively	[154]
	Differentiating distinct minced meat types (beef, mutton, and chicken).	HSI	CNN	94% accuracy	[155]

## Data Availability

Data sharing not applicable.
